# Study protocol for the volume targeted mask ventilation versus pressure ventilation in preterm infants—the VOLT-trial

**DOI:** 10.3389/fped.2025.1743460

**Published:** 2026-01-06

**Authors:** Brenda H. Y. Law, Maryna Yaskina, Peter G. Davis, Graeme Polglase, Michael Dunn, Amit Mukerji, Georg M. Schmölzer

**Affiliations:** 1 Centre for the Studies of Asphyxia and Resuscitation, Royal Alexandra Hospital, Edmonton, AB, Canada; 2 Department of Pediatrics, University of Alberta, Edmonton, AB, Canada; 3 Women and Children’s Health Research Institute (WCHRI), University of Alberta, Edmonton, AB, Canada; 4 Department of Neonatology, The Royal Women’s Hospital, University of Melbourne, Melbourne, VIC, Australia; 5 The Ritchie Centre, Department of Paediatrics, Monash University, Hudson Institute of Medical Research, Melbourne, VIC, Australia; 6 Department of Paediatrics, University of Toronto, Toronto, ON, Canada; 7 Department of Pediatrics, McMaster University, Hamilton, ON, Canada

**Keywords:** infant, extremely preterm, neonatal intensive care, delivery room, respiratory function tests, neonatal mortality

## Abstract

**Background:**

The rapid establishment of gas exchange after birth is vital for survival and long-term health. When newborn infants fail to initiate spontaneous breathing, positive pressure ventilation (PPV) is the cornerstone of respiratory support immediately after birth. The aim of PPV is to inflate the lungs, create a functional residual capacity, deliver an adequate tidal volume (V_T_), facilitate gas exchange, and stimulate breathing, without causing lung or brain injury. In the delivery room, PPV is routinely provided via a pressure-limited device (called a T-Piece resuscitator), where an arbitrary peak inflation pressure (PIP) is set, with the assumption an adequate and safe VT will be delivered. An alternative approach would be using a ventilator to deliver volume-targeted ventilation (VTV), whereby the PIP is adjusted to target a set VT measured by an inline flow sensor. While several trials have evaluated the use of respiratory function monitors to adjust the delivered V_T_ during PPV, no trial has compared PPV with VTV-PPV in the delivery room.

**Methods:**

A randomized trial of VTV-PPV vs. PPV during neonatal resuscitation in preterm infants at birth to assess feasibility of a definitive trial for the intervention. Preterm infants born between 23^0/7^ to 28^6/7^ weeks' gestation will be eligible. Patients will be randomized to either the intervention (VTV-PPV) or the comparator (PPV) during respiratory support in the delivery room. The sample size will be 50 preterm infants. The primary outcome will be percentage of eligible participants (=infants requiring PPV) who have the intervention performed correctly without protocol deviation (=cross over to control group when randomized to VTV-group). Secondary outcomes will include neonatal morbidities (e.g., death, severe brain injury, bronchopulmonary dysplasia; and severe retinopathy of prematurity).

**Discussion:**

The VOLT-trial aims to assess feasibility of VTV-PPV and will address gaps in the evidence regarding the optimal approach to the establishment of ventilation in the delivery room. We aim to use to results of this trial to inform the design of a large multi-centre trial.

**Study Protocol Registration:**

https://clinicaltrials.gov/study/NCT05144724, identifier NCT05144724.

## Introduction

### Background and rationale

#### Limitations of current positive pressure ventilation practices in the delivery room

When newborn infants fail to initiate spontaneous breathing at birth, positive pressure ventilation (PPV) remains the cornerstone of respiratory support immediately (1–3). The purpose of PPV is to inflate and create functional residual capacity in the lung, deliver an adequate tidal volume (V_T_), facilitate gas exchange, and stimulate breathing, all while minimizing lung and brain injury (4). Positive pressure ventilation (PPV) in the delivery room is usually provided via a face mask with a T-piece resuscitator, where peak inflation pressure (PIP) is selected without measuring the actual tidal volume (V_T_) delivered. Instead, adequacy is judged by heart rate and chest wall movement, though studies show healthcare providers often cannot reliably assess chest wall movement (5–7).

#### Tidal volume variability and Its link to lung and brain injury at birth

Preterm infants are susceptible to ventilation-induced lung injury, as the volume difference between functional residual capacity and total lung capacity is small in this population (8). Delivery room studies reported that V_T_ during mask PPV ranges between 0 and 31 mL/kg (6, 9–13). This is concerning as animal studies demonstrated that lung injury was predominantly caused by high V_T_ ventilation (>8 mL/kg) and if V_T_ was controlled to avoid lung over-distention, little or no injury occurred (14–22).

*Similarly, s*tudies in preterm lambs reported that PPV with V_T_ >8 mL/kg causes brain injury through increased inflammation, hemodynamic instability, and oxidative stress (17–19). Polglase et al. showed that preterm lambs ventilated with higher V_T_ (10–12 mL/kg) for the first 15 min after birth had highly variable cerebral oxygenation, as measured by near-infrared spectroscopy (NIRS), compared to preterm lambs ventilated with a protective strategy (V_T_ < 8 mL/kg) (19). Disruption of the blood-brain barrier was observed in 60% of preterm lambs ventilated with high V_T_, as evidenced by vascular protein extravasation, but was not observed in preterm lambs ventilated with a protective strategy. These preclinical findings are supported by observational studies in the delivery room, which reported a four-fold increase in rates of severe intraventricular hemorrhage (IVH) in preterm infants (<29 weeks' gestation) when V_T_ >6 mL/kg was delivered during PPV (23, 24). Therefore, approached which could improve V_T_ delivery might reduce lung and brain injury.

#### Adjusting tidal volume to protect the preterm lung and brain

Randomized trials in the delivery room utilizing a Respiratory Function Monitor (RFM) during PPV, which allows the resuscitator to target V_T_, has been associated with a lower rate of excessive (>8 mL/kg) V_T_ delivery (9–11). A systematic review of randomized trials comparing RFM visible vs. masked demonstrated confirmed that the proportion of infants receiving V_T_ >8 mL/kg can be reduced with an RFM visible (25). However, rates of bronchopulmonary dysplasia were unchanged in all trials. Interestingly, brain injury (defined as any IVH and/or periventricular leukomalacia) can be reduced by 35% and any grade IVH by 32% with an RFM visible (25). Unfortunately, none of the randomized trials was powered to examine differences in brain injury (only reported as secondary outcomes), and therefore these results might have only occurred by chance.

Neonatologists are increasingly careful in the neonatal intensive care unit (NICU) to apply ventilation strategies that are gentle to the lungs (26, 27). Volume-targeted ventilation (VTV), where ventilators algorithmically adjust PIPs to target a set V_T_ as detected through in an inline flow sensor, is routinely used in the NICU (26–28). A meta-analysis of VTV in the NICU showed a reduction in a number of adverse clinical endpoints, including (i) the incidence of pneumothorax (typical RR [95%CI] 0.46 [0.25–0.84], numbers needed to treat [95%CI] 17 [10–100]), (ii) hypocarbia (typical RR [95%CI] 0.56 [0.33–0.96], numbers needed to treat [95%CI] 4 [2–25]), (iii) combined outcome of death or bronchopulmonary dysplasia (typical RR [95%CI] 0.73 [0.57–0.93], numbers needed to treat [95%CI] 8 [5–33]), and (iv) the combined outcome of periventricular leukomalacia (PVL) or grade 3–4 IVH (typical RR [95%CI] 0.48 [0.28–0.84], numbers needed to treat [95%CI] 11 [7–50]) (27). However, despite the general acceptance of VTV in the NICU, clinicians appear less aware that the same gentle approach should be applied to PPV in the delivery room to reduce lung and brain injury immediately after birth [36,43].

#### Unanswered question: does VTV improve outcomes compared to PPV in the delivery room?

Therefore, alternate methods of providing tight V_T_ control during PPV in the delivery room resuscitation of preterm infants is needed, given the complex interaction of mask leak, lung compliance, PIP, and resuscitator skill. Using VTV immediately after birth would provide continuity from the delivery room into the NICU. Tracy et al. compared mask PPV with a T-Piece or VTV during simulated neonatal resuscitation and reported a lower coefficient of variations and lower V_T_ delivery with ventilator driven VTV compared PPV with a T-Piece (29). Similarly, Jain et al. compared VTV via a mechanical ventilator to manual T-Piece PPV and reported improved volume targeting at different compliance settings and a reduction in mask leak during simulated mask ventilation (30). These studies suggest that VTV is feasible at least during animal or simulated neonatal resuscitation. Furthermore, Menakaya et al. compared VTV with a PPV using a Flow-Inflating bag and reported that preterm infants be safely and effectively resuscitated using a ventilator (31). However, no study has compared VTV with T-Piece resuscitator during neonatal resuscitation of preterm infants in the delivery room. Thus, a clinical trial using VTV in the delivery room is urgently needed to determine if (a) this method can be feasibly used in the delivery room and (b) can potentially improve outcomes by reducing bronchopulmonary dysplasia and brain injury in preterm infants.

### Objectives

#### Primary objective

To determine feasibility and safety of a trial comparing VTV-PPV to PPV in extremely preterm infants.

#### Secondary objectives

To evaluate clinical outcomes and HCP experience of the VTV-PPV.

### Primary research question

**Population:** In preterm infants born at 23^0^–28^6^ weeks' gestation is a trial of **Intervention:** mask ventilation using a mechanical ventilator (VTV-PPV) **Comparison:** compared to mask ventilation using a T-Piece Resuscitator (PPV) **Outcome:** feasible during mask ventilation in the delivery room, which will be assessed by percentage of eligible participants (=infants requiring PPV) randomized to the VTV group and receive the allocated intervention without protocol deviation (i.e., without crossover to the control group) Timeline: during the first 10 min after birth?

### Secondary research questions

1. In preterm infants born at 23^0^–28^6^ weeks of gestation, does mask ventilation using a mechanical ventilator compared to mask ventilation using a T-Piece Resuscitator reduce neonatal morbidities (severe brain injury, bronchopulmonary dysplasia; and severe retinopathy of prematurity) and reduce the number of infants requiring intubation in the delivery room by transitioning more successfully to CPAP.
2. What qualitative feedback do HCPs have on the experience of using VTV-PPV in the delivery room?
3. How does VTV-PPV compared with PPV affect subjective workload reported by HCPs performing mask ventilation?

### Trial design

A single centre randomized controlled trial.

## Methods: participants, interventions, outcomes

### Study setting

Royal Alexandra Hospital, Edmonton, Canada, a tertiary perinatal center admitting ∼140 infants born between 23^+0^ to 28^+6^ weeks' gestation annually. The trial is registered on clinicaltrials.gov (https://clinicaltrials.gov/study/NCT05144724) (Table 1).

**Table 1:** Trial timeline.

Time point	Enrolment	Allocation	Post-allocation	Hospital discharge
	Antenatal	At birth	7–10 days of age	
Enrolment				
Eligibility screen	X	X		
Informed consent			X	X
Maternal demographic and pregnancy data			X	
Randomization data		X		
Baseline infant data			X	X
Interventions				
Intervention		X		
Outcome assessments				
Safety Assessment In-hospital Mortality (Part of primary outcome)		X	X	X
Safety Assessment Intraventricular hemorrhage			X	
Completion of admission data				X
Primary Outcome: Feasibility to recruit 50 preterm infants requiring PPV over a 2-year period				X

### Eligibility criteria

#### Inclusion criteria (all must be satisfied)

- Born between 23^0/7^ to 28^6/7^ weeks' gestation based on best obstetrical estimates
- Considered suitable for full resuscitation, i.e., no parental request or antenatal decision to forego resuscitation
- Deferred parental consent post-intervention

#### Exclusion criteria

- Major congenital or chromosomal malformation
- Conditions that might have an adverse effect on breathing or ventilation (e.g., high risk for lung hypoplasia, congenital diaphragmatic hernia)
- Antenatally detected congenital heart disease requiring intervention in the neonatal period
- Antenatally diagnosed Hydrops requiring intervention in the neonatal period
- Neonatal resuscitation initiated before NICU team arrival
- Infants who are born outside of study center and transported to center after delivery

### Consent or assent: who will take informed consent?

Informed parental/guardian consent will be obtained after the study intervention for ongoing data collection. The consent approach of obtaining individual consent after birth for data inclusion in the trial, will strengthen the number being recruited. Furthermore, this approach has been reviewed and approved by our local human ethics review board and is supported by the local Neonatal Family Advisory Care Team. For this important study to be feasible, and enroll a representative sample, an informed consent obtained after the study intervention for ongoing data is important, as obtaining consent prior to delivery can be difficult (32, 33). Guidelines for this approach as laid down by Tri-Council Policy Statement state that any study wishes to use deferred consent needs (34):

a) “A serious threat to the prospective participant requires immediate intervention.” Infants participating in this trial will require respiratory support, which in most cases was unforeseen prior to delivery; hence these infants will all need PPV and using mask ventilation will be therapeutic. Although the pressure limited PPV method (*PPV group*) is the routinely used method at the participating site, it would not be feasible for a neonatal resuscitation study to ask permission prior to delivery from every parent delivering within the participating site.
b) “either no standard efficacious care exists or the research offers a realistic possibility of direct benefit to the participant in comparison with standard care.” The currently used approach in the delivery room is pressure limited PPV. However, in the NICU, VTV is standard of care for providing mechanical ventilation, not for resuscitation on NICU. The available animal evidence and limited neonatal data suggest that the interventional approach has the potential to improve standards of care.
c) “either the risk is not greater than that involved in standard efficacious care, or it is clearly justified by the prospect for direct benefits to the participant.*”* Resuscitation is therapeutic and the currently available evidence suggest that the interventional approach (*VTV-PPV group*) has no higher risk of harm compared to the current standard of care (*PPV group*). Furthermore, the intervention approach is routine standard practice in the NICU, and has been shown to decrease combined outcomes of death/bronchopulmonary dysplasia and brain injury.
d) “the prospective participant is unconscious or lacks capacity to understand the risks, methods and purposes of the research project.” A person in labor cannot give a valid informed consent to a research study. Thus, the parents will be informed as soon as possible after stabilization of the infant about the study and asked to consent to the use of data that have been collected on their child.
e) “third party authorization cannot be secured in sufficient time, despite diligent and documented efforts to do so;” The parents will be informed as soon as possible after birth about the study and asked to consent to the use of data that have been collected on their child.
f) *“*No relevant prior directive by the participant is known to exist.”
g) There is an increasing use of the deferred consent approach within delivery room research. The steering committee of this application has ample experience with using this approach during neonatal resuscitation studies.

### Consent or assent: ancillary studies

Parents of participating infants will be asked if they agree to use of their data should they choose to withdraw from the trial. Participants will also be asked for permission for the research team to share relevant data with people from the Universities taking part in the research where relevant. This trial does not involve collecting biological specimens for storage.

## Interventions

### Choice of comparators

In the delivery room, PPV is routinely provided via a T-Piece resuscitator, where a PIP is arbitrarily chosen, with the assumption it will deliver an adequate V_T_. However, the delivered V_T_ is not measured and therefore PIP is not adjusted to optimize V_T_ delivery. While a lung-protective strategy must start immediately after birth, this has not been studied in detail. Lung compliance and the corresponding PIP needed to deliver an appropriate V_T_ vary between infants depending on gestational age, disease state and delivery mode (C-section vs. vaginal). In addition, the optimal PIP and V_T_ for an individual infant change in the first minutes after birth as lung fluid is cleared and the lung aerated. Therefore, relying on a fixed PIP and subjective assessment of chest rise may result in harm by either under- or over-ventilation. It may be beneficial to measure and adjust the V_T_ delivered during PPV in the minutes after birth, especially in very preterm infants at the greatest risk of lung and brain injury. Delivery room studies reported that V_T_ during PPV ranges between 0 and 31 mL/kg, which is concerning as animal studies reported that lung injury was predominantly caused by high V_T_ ventilation (>8 mL/kg). This led to the utilization of RFMs during PPV to target V_T_. Three trials compared an RFM visible to adjust the delivered V_T_ to RFM masked, which resulted in a reduction of V_T_ >8 mL/kg being delivered, but failed to reduce rates of bronchopulmonary dysplasia. Therefore, alternate methods of providing tight V_T_ control during PPV in the delivery room resuscitation of preterm infants is needed. In the NICU, VTV ventilators which algorithmically adjust PIPs to target a set V_T_ are routinely used. A meta-analysis of trials comparing VTV with pressure guided ventilation in the NICU showed a reduction in pneumothorax, combined outcome of death or bronchopulmonary dysplasia, combined outcome of PVL or grade 3–4 IVH. However, despite the general acceptance of VTV in the NICU, this approach is not used in the delivery room to reduce lung and brain injury immediately after birth. The trials will compare VTV-PPV with PPV during respiratory support immediately after birth to assess the feasibility of VTV-PVV.

### Intervention description

#### Treatment arms

##### PPV group

**Figure 1:**
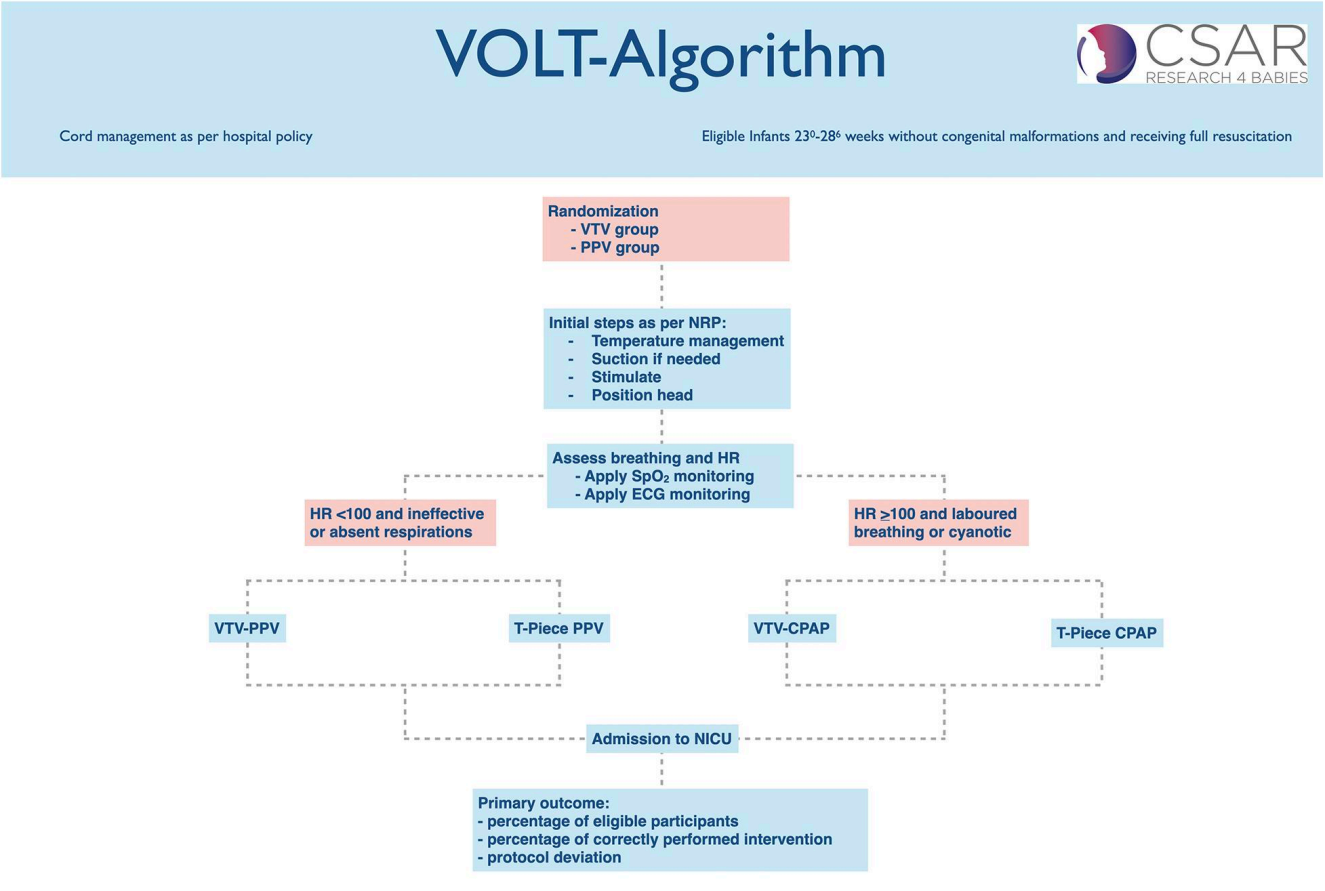
VOLT study algorithm.

The clinical team will proceed through all steps of the neonatal resuscitation algorithm, as per current neonatal resuscitation guideline (1–3) and local hospital policy (standard hospital practice guideline). Mask ventilation in the delivery room will be provided as per local hospital policy (standard hospital practice guideline). At the Royal Alexandra Hospital, mask ventilation is delivered with a T-piece device with initial settings of PIP of 24 cmH_2_O, a peak expiratory pressure PEEP of 6 cmH_2_O, a max PIP of 40 cmH_2_O and a gas flow rate of 8–10 L/min. Resuscitators are trained to use a ventilation rate of 40–60 inflations/min. The clinical team will determine if/when ventilation pressures (both PEEP and PIP) should be increased, as per local hospital policy (standard hospital practice guideline), using clinical assessments (heart rate, oxygen saturation, auscultation, chest raise) (Figure 1).

##### VTV-PPV group

Initial ventilation settings for VTV-PPV will be a VT of 5 mL/kg, a ventilation rate of 50 inflations/min, a maximum PIP of 40 cmH_2_O, and a PEEP of 6 cmH_2_O. For VTV-PPV, a size 9.0 endotracheal tube connector will be inserted into the facemask to allow secure attachment of the flow sensor and ventilator circuit. Ventilation will be delivered using PC-CMV with Volume Guarantee, beginning at 5 mL/kg and adjustable up to 6 mL/kg if required. The trigger sensitivity will be set at 0.2 L/min to enable infant-triggered breaths, and the inspiratory time will be set at 0.3 s, with the option to increase to 0.5 s based on clinical response. To minimize leak, clinicians will use a two-hand mask-hold technique, as the ventilator tubing is heavier than a T-piece and may otherwise create downward pull. The clinical team will proceed through all steps of the neonatal resuscitation algorithm, as per current neonatal resuscitation guidelines (1–3) and local hospital policy but will replace standard pressure-limited PPV with VTV-PPV in the intervention group. The clinical team will determine if and when V_T_, ventilation rate, and/or PEEP should be increased, using clinical assessments (heart rate, oxygen saturation, auscultation, chest rise). In addition, the clinical team will be trained to troubleshoot ventilator alarms such as low minute ventilation (e.g., airway obstruction) and disconnect alarms (e.g., mask leak) (Figure 1). No CO_2_ monitoring (end-tidal or transcutaneous) will be used due to feasibility limitations in the delivery room.

#### Duration of treatment period

The study intervention will be applied for the first 10 min after birth.

### Criteria for discontinuing or modifying allocated interventions

In any cases where the resuscitation team believes, that VTV-PPV is not working, the resuscitation team can revert to standard hospital practice guideline of administering PPV using a T-Piece resuscitator at any time.

If HR <60 at any time despite 30 s of effective ventilation, the resuscitation guidelines state to use an alternate airway and start chest compression. At that time, the VTV-PPV approach must be abandoned and a T-Piece must be used.

### Strategies to improve adherence to interventions

Training logs and continuing engagement during daily clinical huddles to remind about the study.

### Relevant concomitant care permitted or prohibited during the trial

Other than providing PPV with either a ventilator (intervention) or a T-Piece (standard of care), all delivery room interventions will follow the center's local hospital policy (standard hospital practice guideline) and the current neonatal resuscitation guidelines (1–3).

### Provisions for post-trial care

Care during the primary hospitalization will adhere to local practice guidelines.

### Outcomes

#### Primary outcome

Percentage of eligible participants (=infants requiring PPV) who have the intervention performed correctly without protocol deviation (=cross over to control group when randomized to VTV-group).

#### Secondary outcomes

Secondary outcomes include:

1. All-cause in-hospital mortality
2. Severe brain injury on cranial ultrasound: Severe grade 3 and 4 intraventricular or intraparenchymal hemorrhage according to Papile (35), periventricular leukomalacia, or ventriculomegaly based on neuroimaging studies (timing and frequency of imaging based on local site practices)
3. Severe retinopathy of prematurity (stage 3 or higher) as defined in the International Classification of ROP, and/or ROP treated with laser, cryotherapy, or intraocular injection therapy (36)
4. Bronchopulmonary dysplasia at 36 weeks corrected age and at 40 weeks corrected age, defined as receiving any supplemental oxygen or any form of respiratory support (including invasive mechanical ventilation, non-invasive ventilation with continuous positive airway pressure, nasal intermittent positive pressure ventilation, or high-flow nasal canula)
5. Total duration of mechanical ventilation via an endotracheal tube in days
6. Discharge home on oxygen
7. Duration of any respiratory support (invasive mechanical ventilation, non-invasive ventilation with continuous positive airway pressure, nasal intermittent positive pressure ventilation, or non-invasive neural assist ventilation or non-invasive high frequency ventilation, or high-flow nasal cannula) in days
8. Duration of supplemental oxygen in days
9. Length of hospital stay in days
10. Rate of intubation in the delivery room
11. Rate of chest compression in the delivery room
12. Rate of pneumothorax (diagnosed by chest x-ray or lung ultrasound) and interventions (e.g., needle drainage or chest drain)
13. Necrotizing enterocolitis, Modified Bell's criteria stage 2 or greater (37)
14. Duration of positive pressure ventilation at birth (within the first 10 min)
15. Resuscitators subjective workload performing mask ventilation, as evaluated by Surgery Task Load Index (SURG-TLX)-questionnaire.
16. Resuscitators qualitative feedback on using VTV-PPV in the delivery room, provided through free text responses.

### Participant timeline

### Sample size

This will be a trial to assess the feasibility of VTV-PPV in the delivery room. Our primary outcome will assess the percentage of eligible participants (=infants requiring PPV) who have the intervention performed correctly without protocol deviation (=cross over to control group when randomized to VTV-group). We aim to recruit a sample of 50 infants (25 per arm) randomized 1:1 to VTV-PPV or PPV. The proposed sample size should be reached within 24 months. This study will be an initial trial for a large multi-center trial.

### Recruitment

The study will be carried out at the Royal Alexandra Hospital, Edmonton, Canada, a tertiary perinatal center with ∼6,000 deliveries annually admitting ∼140 infants born between 23^+0^ to 28^+6^ weeks' gestation annually. Approximately 60% of babies born between 23 and 28 weeks' gestation require PPV at birth. The study site has a long history of conducting randomized trials and has the proven capability of enrolling the required number of infants. The number of infants being recruited will be 50, as per sample size. The overall recruitment period is a maximum of 24 months. There will be 50 infants recruited in Canada.

## Assignment of interventions

### Allocation

#### Sequence generation

The Biostatistics Unit at Women and Children's Health Research Institute (WCHRI), University of Alberta, Edmonton, Canada, will prepare the randomization schedule. Before trial initiation, a statistician will generate the 1:1 allocation sequence with computer-generated random numbers, which a second statistician will independently validate.

#### Concealment mechanism

The site's allocation sequence is password-protected, with access restricted to the independent statistician.

#### Implementation

Before the start of the trial, the statistician will use computer-generated random numbers to prepare the allocation sequence by producing the codes and allocation table. Clinical staff attending neonatal deliveries will open an envelope with the group assignment and will enroll participants.

### Assignment of interventions: blinding

#### Who will be blinded

Blinding will not be feasible in the delivery room, as the healthcare providers will use two different ventilation devices including the VN 500 Ventilator (VTV-PPV Group) and the Neopuff T-Piece (PPV Group). The outcome assessor will be unaware of the group allocation. This blinding will be maintained until the data is locked for the final analysis, which will be performed blinded to group analysis and then un-blinded.

#### Procedure for unblinding if needed

Members of the Data Safety Monitoring Board (DSMB) will access unblinded treatment allocations to determine causality for any severe adverse events (SAEs) or other serious trial-related events.

### Data collection and management

#### Plans for assessment and collection of outcomes

The VOLT investigators and research nurses will collect data from maternal and infant paper or electronic medical records. Data will be entered into a REDCap™ (Vanderbilt University) electronic database, designed and managed at the University of Alberta. REDCap is a secure, web-based application that supports data capture for research studies, including database management, survey design, and audit trails to ensure data integrity.

#### Plans to promote participant retention and complete follow-up

We are anticipating a <5% loss to follow-up until hospital discharge. The center has conducted more than 20 neonatal trials in the delivery room with lost-to-follow-up rates for the primary outcome of <5%.

#### Data management

VOLT investigators and research nurses will collect maternal and infant data from paper or electronic records and enter it into REDCap™ (Vanderbilt University), a secure, web-based research database designed and managed by the University of Alberta (38, 39).

#### Confidentiality

Participant data will be handled in compliance with applicable data protection and privacy regulations. All data will be securely stored, with electronic records accessible only to authorized research team members via password protection. Data will be de-identified, and participant anonymity will be maintained in all scientific publications and presentations.

#### Biological specimens

There will be no biological specimens collected.

## Statistical methods

### Statistical methods for primary and secondary outcomes

WCHRI will handle, verify, and analyze VOLT-trial data, with statistical methods aligned to standard randomized trial practices. Results will be reported in compliance with CONSORT guidelines.

The primary analysis will be conducted using an “intention-to-treat” approach. The primary analysis will focus on feasibility defined as percentage (75% or greater) of eligible participants (=infants requiring PPV) who have the intervention performed correctly without protocol deviation (=cross over to control group when randomized to VTV-group). Analysis of secondary outcomes will include the above-mentioned secondary outcomes using descriptive statistics. Summary statistics will be presented for baseline and clinical characteristics; continuous data by mean, two-sided 95%CI of the mean, standard deviation, median, interquartile range (first and third quartiles), minimum and maximum. Categorical data will be presented by absolute and relative frequencies. The clinical characteristics and outcome parameters will be compared using Student's *t*-test for parametric and Mann–Whitney *U*-test for nonparametric comparisons of continuous variables, and *χ*^2^ for categorical variables. The analysis will be 2-sided and *p*-value <0.05 will be considered statistically significant.

### Interim safety analyses

The DSMB will conduct one interim safety analyses throughout the trial to assess in-hospital mortality and SAEs after 24 (50%) infants recruited.

### Methods for additional analyses (e.g., subgroup analyses)

At the end of each intervention, the clinician's workload will be assessed using the Surgery Task Load Index (SURG-TLX)-questionnaire (40). The SURG-TLX- questionnaire is a multidimensional workload measure to assess the impact of six various sources of stress on the perceived demands of trained healthcare professionals. These six aspects are then combined into a total workload score.

The SURG-TLX- questionnaire will assess:

1. Task complexity (How complex was the procedure?)
2. Physical demands (How physically fatiguing was the procedure?)
3. Mental demands (How mentally fatiguing was the procedure?)
4. Distraction (How distracting was the operating environment?)
5. Situational stress (How anxious did you feel while performing the procedure?)
6. Temporal demands (How hurried or rushed was the pace of the procedure?)

Furthermore, clinicians will be asked about their experience of using VTV-PPV by completing a short survey to solicit their feedback on switching between CPAP and PPV with the ventilator, not directly manipulating airway pressures during VTV-PPV, and performing ventilation corrective steps during VTV-PPV.

### Analysis population and missing data

There will be no analysis for missing data will be reported and sensitivity analyses considered.

### Plans to give access to the full protocol, participant level-data and statistical code

Trial information is publicly available on ClinicalTrials.gov (NCT05144724) and the Research4Babies website. The protocol will be shared and submitted for publication, and the de-identified VOLT dataset will be released 6 months after the primary outcome is published; data requests can be emailed to georg.schmoelzer@me.com and will be decided by the VOLT Trial Steering Committee.

## Oversight and monitoring

### Composition of the coordinating center and trial steering committee

The trial management team is based at the Royal Alexandra Hospital, Edmonton, Canada, includes the Principal Investigators (GMS), and the Trial coordinators (Caroline Fray and Erin Perla), and meets weekly (Table 2).

**Table 2:** Composition of the coordinating center and trial steering committee.

Prof Georg M. Schmölzer (Nominated Principal Investigator)	Centre for the Studies of Asphyxia and Resuscitation, Edmonton, Canada
	University of Alberta, Edmonton, Canada
Assistant Prof Brenda Law (Principal Investigator)	Centre for the Studies of Asphyxia and Resuscitation, Edmonton, Canada
	University of Alberta, Edmonton, Canada
Dr. Maryna Yaskina (Senior Biostatistician)	Women and Children's Health Research Institute, University of Alberta, Edmonton, Canada
Prof Peter G. Davis (Principal Investigator)	Melbourne University, Melbourne, Australia
Prof. Graeme Polglase (Principal Investigator)	Monash University, Melbourne, Australia
Michael Dunn (Principal Investigator)	University of Toronto, Toronto, Canada
Amit Mukerji (Principal Investigator)	McMaster University, Hamilton, Canada
Caroline Fray (Trial coordinator)	Centre for the Studies of Asphyxia and Resuscitation, Edmonton, Canada
Erin Perla (Trial coordinator)	Centre for the Studies of Asphyxia and Resuscitation, Edmonton, Canada

### Trial steering committee

The Trial Steering Committee detailed below meets approximately quarterly, chaired by GMS.

### Composition of the data monitoring committee, its role, and reporting structure

The DSMB, composed of three independent members (Chair, neonatal clinician, and biostatistician), is guided by a pre-finalized Charter to safeguard participants, monitor trial conduct, advise investigators, and oversee interim safety reviews. It will meet about every six months and for interim safety analyses, with no pre-specified stopping criteria (Table 3).

**Table 3:** Data Safety Monitoring Board.

Prof Anup Katheria	Chair	San Diego, USA
Prof Anne Solevåg	Independent Expert	Oslo, Norway
Ben Vandermeer	Statistician	Edmonton, Canada

### Adverse event reporting and harms

Safety reporting from the VOLT-Trial will follow standards from the University of Alberta Human Research Ethics Board and the Tri-Council Policy Statement: Ethical Conduct for Research Involving Humans (34).

Pre-defined SAE are:

- Death in the delivery room
- Death in the Neonatal Intensive Care Unit

### Auditing

The Ethics Committee will review trial conduct annually, while the independent DSMB will meet every six months for trial oversight and interim safety analyses. The Trial Steering Committee will convene quarterly to monitor conduct throughout the study.

### Plans for communicating important protocol amendments to relevant parties

Since trial initiation, there have been no major protocol amendments. Minor modifications have been submitted for approval to the relevant ethics committees and subsequently distributed and communicated to all participating sites.

## Dissemination plans

### Trial results

Trial results will be shared at national and international conferences, submitted to high-impact journals, and disseminated through media and social media. A lay summary, developed in collaboration with the Canadian Premature Babies Foundation, will be provided to all participating families.

## Discussion

The VOLT-trial will evaluate the feasibility of using VTV-PPV in the delivery room to reduce brain injury and improve outcomes in extremely preterm infants. Findings will address evidence gaps in initial ventilation at birth and inform a future large-scale definitive randomized trial.

## Trial status

The current Protocol version is 3.5, dated October 4, 2022. Recruitment began in October 2024 at Royal Alexandra Hospital, Edmonton. Recruitment is expected to be completed in 2026 with results expected in 2027.
